# SynTReN: a generator of synthetic gene expression data for design and analysis of structure learning algorithms

**DOI:** 10.1186/1471-2105-7-43

**Published:** 2006-01-26

**Authors:** Tim Van den Bulcke, Koenraad Van Leemput, Bart Naudts, Piet van Remortel, Hongwu Ma, Alain Verschoren, Bart De Moor, Kathleen Marchal

**Affiliations:** 1 ESAT-SCD, K.U.Leuven, Kasteelpark Arenberg 10, B-3001 Heverlee, Belgium; 2 ISLab, Dept. Math. and Comp. Sc., University of Antwerp, Middelheimlaan 1, B-2020 Antwerpen, Belgium; 3 Dept. of Genome Analysis, German Research Center for Biotechnology, Mascheroder Weg 1, D-38124 Braunschweig, Germany; 4 CMPG, Dept. Microbial and Molecular Systems, K.U.Leuven, Kasteelpark Arenberg 20, B-3001 Heverlee, Belgium

## Abstract

**Background:**

The development of algorithms to infer the structure of gene regulatory networks based on expression data is an important subject in bioinformatics research. Validation of these algorithms requires benchmark data sets for which the underlying network is known. Since experimental data sets of the appropriate size and design are usually not available, there is a clear need to generate well-characterized synthetic data sets that allow thorough testing of learning algorithms in a fast and reproducible manner.

**Results:**

In this paper we describe a network generator that creates synthetic transcriptional regulatory networks and produces simulated gene expression data that approximates experimental data. Network topologies are generated by selecting subnetworks from previously described regulatory networks. Interaction kinetics are modeled by equations based on Michaelis-Menten and Hill kinetics. Our results show that the statistical properties of these topologies more closely approximate those of genuine biological networks than do those of different types of random graph models. Several user-definable parameters adjust the complexity of the resulting data set with respect to the structure learning algorithms.

**Conclusion:**

This network generation technique offers a valid alternative to existing methods. The topological characteristics of the generated networks more closely resemble the characteristics of real transcriptional networks. Simulation of the network scales well to large networks. The generator models different types of biological interactions and produces biologically plausible synthetic gene expression data.

## Background

Recent technological advances have made the application of high throughput assays, such as microarrays, common practice. The ability to simultaneously measure the expression level of a large number of genes, makes it possible to take a system-wide view of the cell.

Developing reliable data analysis methods that infer the complex network of interactions between the various constituents of a living system based on high throughput data, is a major issue in current bioinformatics research [1]. Because data on transcriptional regulation are most accessible, much effort goes to the develoment of algorithms that infer the structure of transcriptional regulatory networks (TRNs) from this data [2-6].

Gaining statistical knowledge about the performance of these algorithms requires repeatedly testing them on large, high-quality data sets obtained from many experimental conditions and derived from different well-characterized networks. Unfortunately, experimental data sets of the appropriate size and design are usually not available. Moreover, knowledge about the underlying biological TRN is often incomplete or unavailable.

As a consequence, validation strategies applied to experimentally obtained data are often limited to confirming previously known interactions in the reconstructed network. However, using such an approach, false positive interactions are for example not penalized. Indeed, assessing the relevance of predicted interactions that have not been experimentally confirmed, is infeasible. Secondly, the algorithm can usually only be applied to data from a single network, which complicates algorithm design and validation.

Due to these limitations of real experimental data, the use of simulated data for benchmarking structure learning algorithms is gaining interest. The term *network generator *is used to denote a system that generates synthetic networks and simulated gene expression data derived from these networks. A synthetic network consists of a topology that determines the structure of the network and a model for each of the interactions between the genes.

Different approaches have been used to create network topologies. The generation of small networks is often based on detailed handcrafted topologies [7-9]. For producing topologies of large networks comprising thousands of nodes, random graph models have been used [10,11]. The latter models create graphs that share one or more statistical properties, such as scale-free [12] and small-world [13] properties, with known regulatory networks, in an attempt to approximate biological reality.

For simulation of the regulatory network, the interactions between the genes need to be quantitatively modeled. Several models have been proposed for this purpose, including Boolean [14,15], continuous [7,9,11] and probabilistic [11] approaches. Most current network simulators [7-11] use a set of ordinary differential equations (ODE's). The choice of a numerical solution method, which depends on the desired precision and the specific form of the set of ODE's, can lead to scalability problems. The time complexity of numerically solving a set of ODE's for a given time period, in function of the number of genes, varies between linear and cubic complexity, which makes simulation of large networks computationally difficult.

We propose a network generator that copes with some of the limitations of previous implementations. Instead of using random graph models, topologies are generated based on previously described source networks, allowing better approximation of the statistical properties of biological networks. The computational performance of our simulation procedure is linear in function of the number of genes, making simulation of large networks possible.

The tool, called ***SynTReN ***(**Syn**thetic **T**ranscriptional **Re**gulatory **N**etworks), has been implemented in Java and is available for download as additional file 3: *SynTReN.zip*.

## Results

### Overview

**Figure 1:**
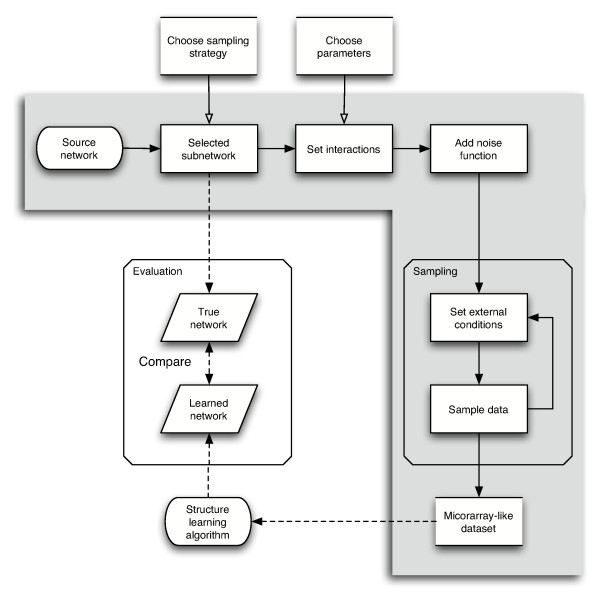
**Network generator overview**. Network generator overview. The shaded area gives a schematic representation of the data generation process. Dashed arrows show how generator output fits in an algorithm validation strategy.

The network generator produces synthetic transcriptional regulatory networks (TRNs) and corresponding microarray data sets. In these networks, the nodes represent the genes and the edges correspond to the regulatory interactions at transcriptional level between the genes. Figure 1 shows the flow of the data generation process comprising three essential steps. In a first step, a network topology is selected from a known source network using either of two selection strategies. In a second step, transition functions and their parameters are assigned to the edges in the network. In the third step, mRNA expression levels for the genes in the network are simulated under different conditions. After optionally adding noise, a data set representing normalized and scaled microarray measurements is obtained. A more detailed explanation is given in Section *Methods*.

**Figure 2:**
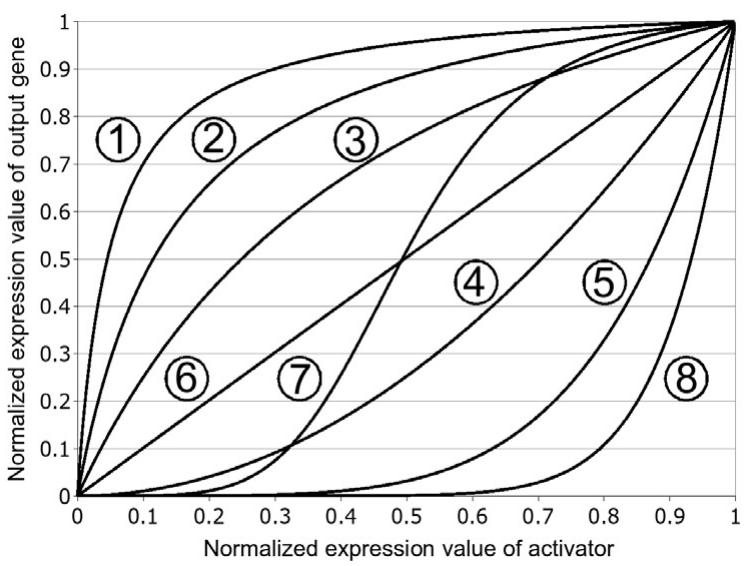
**Interaction types for one activator**. Examples of interaction functions for one activator and different combinations of the kinetic parameters K and h. Parameter tuples (K,h) are (0.05,1); (0.15,1); (0.5,1); (10,2); (10,5); (100,1); (0.5,5); (100,10) for functions 1 to 8 in ascending order.

To evaluate to what extent our approach compares to previous approaches in generating networks with topological characteristics of true TRNs, we used well established deterministic and informative measures that can be subdivided in two distinct categories, each of which addresses different aspects of the network structure: high-level (global) measures and low-level (local) measures.

The high-level measures like average clustering coefficient and average path length, depend on knowledge of the complete network structure while the low level measures are derived from local network properties such as for instance the marginal degree distributions [13,16]. Rigorous definitions are given in additional file 1: *addendum.pdf*.

Biological TRNs have specific common structural properties: the small world property [12], indicating a short average path length between any two nodes and the scale-free property [13], indicating the degree distribution of the nodes of the network follows a power law. True biological TRNs also contain specific structural motifs that are statistically overrepresented as compared to random graphs of the same in- and outdegree [17,18] (e.g. feed forward loop). Synthetic TRNs have been generated using different types of random graph models [10,11], such as Erdös-Rényi [19], Albert-Barabási [13] and Watts-Strogatz [12] models. These models generate graphs with one or more topological properties observed in biological TRNs. Unlike previous approaches, we generate network topologies by selecting subgraphs from a previously described biological source network (*E. coli *[18,20] or *S. cerevisiae *[21]).

### Random network models

To validate our approach, a series of synthetic networks is generated both by using different types of random graph models (Erdös-Rényi (ER), Watts-Strogatz or small-world (SW), Albert-Barabási (AB) and directed scale free (DSF) random graph models) and by selecting subgraphs according to the methods described in Section *Network topology*. To obtain representative sets of networks for the given models, a sweep was done across a large range of possible parameter settings for the tested models. The topological properties of each of these networks are compared to these of the complete *E. coli *and *S. cerevisiae *networks.

**Figure 3:**
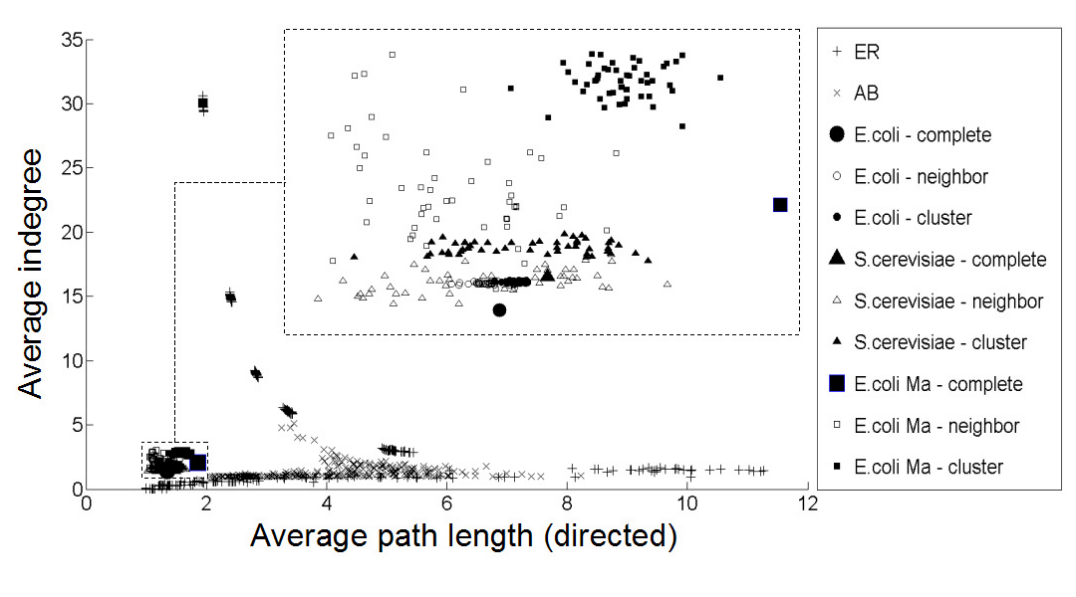
**Topological properties of ER and AB random graphs and subselected networks of 300 nodes and the complete biological networks**. Average indegree versus average directed path length for ER and AB random graphs of 300 nodes and biological networks. Biological networks are the complete *E. coli *(both networks described [18] and [20]) and *S. cerevisiae *network, indicated by the suffix "-complete". Subnetworks containing 300 nodes were created by both the neighbour- and cluster-addition method, indicated by the suffixes "-neighbor" and "-cluster" respectively. The region of the biological networks is enlarged in the upper right corner of the figure. ER and AB random graphs exhibit a phase transition [28,29]. For low connectivity, often no path exists between several pairs of nodes and many path lengths are therefore infinity. These are not considered for calculating the average path length, which therefore appears small because it is calculated from the few short paths that are present. When increasing the *p*-value (the probability of having an edge), the paths are increasingly made up of more edges until a point is reached where the graph starts forming one giant network. Adding more edges then increases the density of the graph connections, resulting in a decrease of the average path length. ER: Erdös-Rényi, AB: Albert-Barabási.

**Figure 4:**
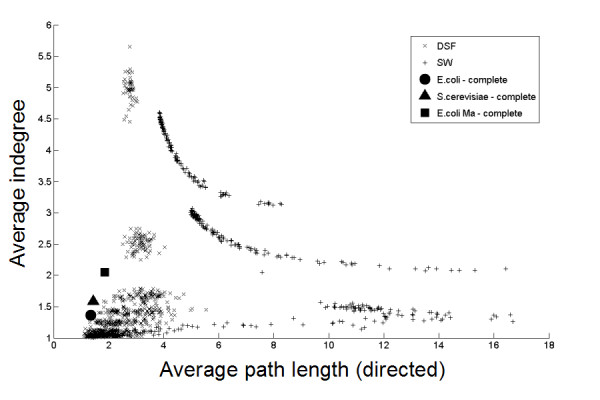
**Topological properties of DSF and SW random graphs of 300 nodes and the complete biological networks**. Average indegree versus average directed path length for DSF and SW graphs of 300 nodes and biological networks. Biological networks are the complete *E. coli *(both networks described by [18] and [20]) and *S. cerevisiae *network [21]. SW: Small world (Watts-Strogatz, [12]), DSF: Directed scale free (Bollobá, [22]).

In Figure 3 and Figure 4, the average indegree is plotted versus the average directed path length to illustrate the different characteristics of the random graph models, the previously described TRNs and the selected subnetworks.

Figure 3 shows that it is not possible to choose the parameters for Erdös-Rényi and Albert-Barabási random graph models such that both the average directed path length and average indegree are simultaneously close to the values of biological TRNs, although they can resemble biological networks for a single topological measure. Similar results are obtained for evaluated topological characteristics other than average indegree and average directed path length, such as average clustering coefficient, average out-degree, average undirected path length, ... (results not shown).

From Figure 4, a similar conclusion can be drawn for SW networks. They can resemble biological networks for a single topological measure, but not for several measures simultaneously. DSF graphs [22] can however resemble biological networks with respect to both average directed path length and average indegree for well-chosen parameter settings. Again, similar results are obtained for other topological characteristics (results not shown).

### Subnetwork selection methods

Figure 3 (inset) shows that, irrespective of the subnetwork selection method used, the properties of the selected subnetworks more closely approximate those of the source TRNs than the properties of the tested random graphs do. Mutually comparing the subnetwork selection methods, it is clear that the cluster addition method generates networks that are closer to the source network than the networks generated with the neighbor addition method. These observations also hold for topological characteristics other than average directed path length and average indegree (results not shown).

To evaluate the change in topological characteristics in function of the number of nodes in the subnetwork, networks of different sizes were selected using both methods (See additional file 2: *subgraphselection.png*).

The neighbor addition method shows more variation for the median indegree compared to the cluster addition method. This is not surprising since adding a node and all of its children, as is done in the cluster addition method, better preserves the median indegree than adding a single node, as done in the neighbor addition method.

### Simulated expression data

**Figure 5:**
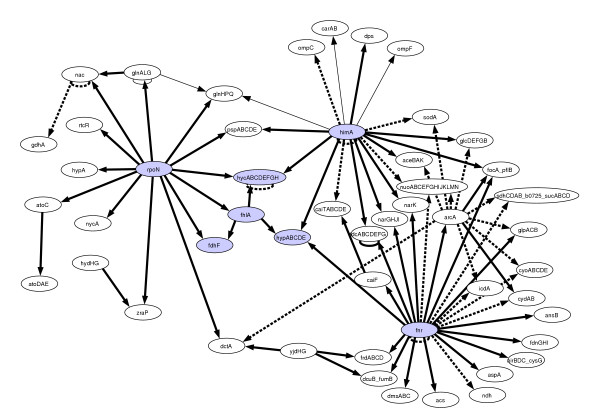
**Example network with 50 nodes**. Randomly chosen example network with 50 nodes, using cluster addition method for subnetwork selection and selection from *E. coli *network. Dashed edges indicate activation, full edges indicate repression.

Figure 5 gives a representative example of a network topology of 50 genes obtained by selection from the *E. coli *source network [20] using the cluster addition method. From this network, gene expression data was generated for 100 simulated microarray experiments.

Three input genes were defined (*g1*, *g2*, *g3*) for which setting the expression values corresponds to changing external conditions in a microarray experiment. Distinct external conditions were thus mimicked by randomly choosing the expression values between 0 and 1 for each experiment.

**Figure 6:**
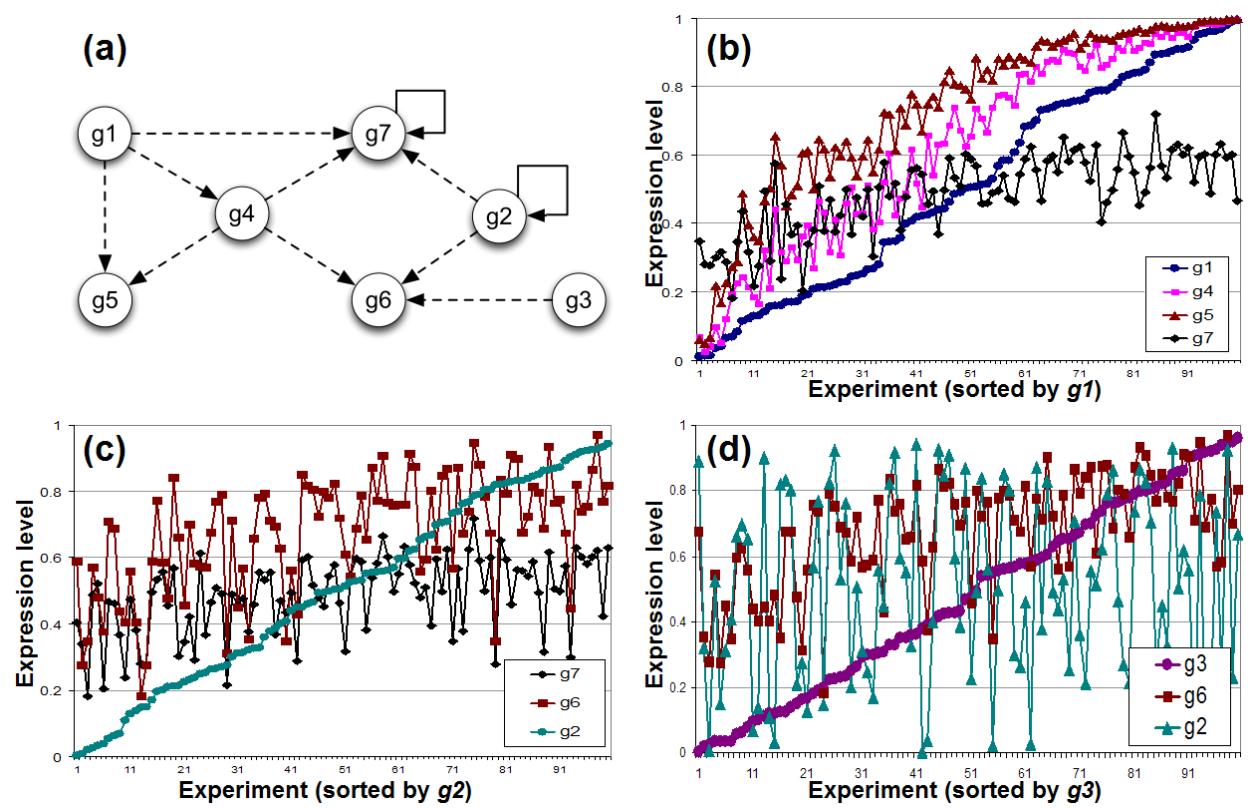
**Simulation results for a subset of genes of the example network**. Simulation results for a subset of genes of the example network (labeled *g1 *to *g7*). This subnetwork has three input genes and contains a repressor feedback loop for *g7*. The X-axes shows the different conducted experiments, which are sorted according to the expression value of each of the input genes. The Y-axes shows the normalized expression values for the genes directly regulated by the input genes, except for (d) where the expression value for *g2 *is also shown.

In Figure 6, part of the simulation results of the example network are shown. The selected part of the example network (also indicated by the shaded nodes in Figure 5) is shown in (a). Figure 6(b),(c) and 6(d) show how each of the input genes affects its direct children. E.g. *g1 *has a strong effect on both *g4 *and *g5*, but a less pronounced effect on the expression level of *g7*, since *g7 *has a repressor feedback loop and is also stimulated by another input gene *g2*. In Figure 6(d), the expression levels of *g2 *are also added to illustrate the relation between two independent genes like *g3 *and *g2*.

## Discussion

Because of the urgent need for well characterized datasets to benchmark network inference methodologies, the use of simulated data has become essential. Therefore, we have developed a generator that produces simulated gene expression data that resembles biological data, but at the same time is computationally tractable for large networks. To more closely approximate the topological characteristics of biological networks, we propose a novel way of generating topologies, i.e. by selecting subnetworks from described biological networks.

Subnetwork selection results in networks that resemble previously described networks across a range of tested topological characteristics. In this respect, the *cluster addition *method performs better than the *neighbor addition *method. Moreover, our selection strategy outperforms several alternative approaches based on random graph models. Our results show that the properties of synthetic topologies created by random graph models depend largely on the input parameters used. Properties of such random graphs generally differ from those of known biological networks in at least one topological measure. Only with the more sophisticated models, such as the directed scale free graph model [22], and given a specific set of input parameters, were we able to generate topologies that resemble previously described TRNs for the complete set of evaluated characteristics.

Our method offers a valid alternative over generating networks using random graph models. However, using previously characterized TRNs as a source of synthetic network topologies implies a dependency on available knowledge about these networks. Obviously, not all interactions are known and some described interactions might be false positives. Moreover, previously described networks might be biased towards well studied pathways. The more accurate these characterized networks will become, the higher the quality of the selected topologies will be.

While DSF networks seem very similar to biological networks under specific parameter settings, it should be noted that finding the optimal set of parameters for the DSF model to create graphs that resemble biological networks is not trivial. Moreover, it is possible that some subtle characteristics of real networks, such as network motifs [17] or joint degree distributions [16], are not captured in the measured properties. Also, as knowledge about true transcriptional networks improves, there is no guarantee that adequate parameters for the DSF networks will still exist. In these cases, selecting subnetworks from known biological networks ensures that the generated topologies will still sufficiently resemble their biological source networks.

The choice of equations based on Michaelis-Menten and Hill kinetic equations to model regulatory interactions, allows a variety of interaction types likely to occur in real biological systems [11,23], ranging from a nearly linear behavior to very steep interactions. Although in genuine networks all dynamic interactions are coupled, we assumed that the steady-state kinetics of the complete network of uncoupled equations are comparable to those of the coupled set of equations. Also, all individual transcription rates are assumed to be in a steady-state regime. In contrast to alternative simulators that solve coupled differential equations, introducing these simplifications made simulating large networks comprising thousands of genes computationally tractable.

Several parameters controlling the gene network generation and sampling process are user-definable in order to generate data sets of increasing level of difficulty. This allows thorough benchmarking of inference algorithms, while low level parameters (such as the kinetic parameters of the enzyme kinetic equations) are automatically chosen from predefined distributions.

The tool can be further improved in a number of different aspects. Firstly, the topologies of the source networks are a key aspect of the performance of the generator. The availability of more accurate data for the gene regulatory networks of *E. coli *and *S. cerevisiae*, will result in more accurate network topologies. The availability of large regulatory networks for organisms of which the network topology differs significantly from that of *E. coli *or *S. cerevisiae *will definitely increase the reliability of our generator for organisms other than *E. coli *and *S. cerevisiae*. Secondly, as more empirical data becomes available, a better estimate of the transition functions and their parameters can be given [24].

Our system can be extended in natural way with additional data sources (e.g. ChIP-chip data, protein-protein interaction data). This data could be sampled from real biological data sets in a way similar to the procedure that was described for the topology selection.

## Conclusion

Our results show that it is quite difficult to create synthetically generated networks that are topologically similar to the biological networks derived for *E. coli *and *S. cerevisiae*, using random graph models. The characteristics of the generated networks were assessed using a set of topological measures and were shown to be very dependent on the input parameters of the random graph model. Only more sophisticated models, such as the DSF graph model [22] are capable of generating networks that resemble the known TRNs for the set of evaluated characteristics, and only given a specific set of input parameters.

The strategy of selecting connected substructures from a source network produced graphs that quite closely resemble the characteristics of a real TRN, provided the subgraphs are sufficiently large. The *cluster addition *method performed better than the *neighbour addition *method in this respect.

The generation of gene expression data scales linearly as a function of the number of nodes and therefore allows fast simulation of large networks comprising thousands of genes.

In this study we focused on the construction of a generator of transcriptional networks and corresponding normalized microarray data. Its primary goal is to offer benchmark datasets for testing and optimizing network inference methods. Both a reasonable approximation of biology and scalability to large networks were major design considerations. By selecting topologies from known networks, modeling interactions by kinetic equations and introducing some major simplifications, we developed a flexible framework that generates benchmark datasets of varying levels of complexity.

## Methods

### Network topology

In this section the process of generating the network topology and corresponding gene interactions is described.

#### Selection of subnetworks

To generate a network topology that resembles a true TRN as closely as possible, network structures are selected from previously described biological networks. The topologies of the well-described model organisms *E. coli *[18,20] and *S. cerevisiae *[21] are used as source graphs (an overview of their main properties is given in Table 1). The choice of source network is user-definable. A single source network at a time is used when generating networks. Two different strategies to select a connected subgraph from a source graph are implemented. In a first strategy, called *neighbor addition*, a randomly selected node is chosen as initial seed. Subsequent nodes are added in an iterative process. Only randomly selected nodes that have at least one connection to the current graph are retained. In an alternative strategy, called *cluster addition*, a randomly selected node and all of its neighbors are selected as initial graph. In each iteration, a randomly selected node and all of its neighbors are added to the graph. Similarly, only nodes that have at least one connection to the current graph are retained. Because of their presence in the original source network, cycles (e.g. feedback loops) can also be encountered in the generated topology.

**Table 1:** Properties of *E. coli *and *S. cerevisiae *networks. This table compares the charactistics of *E. coli *and *S. cerevisiae *source networks which are used in the creation of subnetworks. The extended *E. coli *network described by Ma et al. [20] has over three times more nodes than the *E. coli *network described by Shen-Orr et al. [18]. The average path length is longer for this network and the average clustering coefficient is higher. For rigorous definitions of the measurements see additional file 1: *addendum.pdf*.

**Network properties**	***E. coli *[18]**	***E. coli *[20]**	***S. cerevisiae***
Number of nodes	423	1330	690
Number interactions	578	2724	1094
Average directed path	1.36	1.85	1.44
Average clustering coefficient	0.085	0.20	0.047

#### Background network

For a real biological microarray experiment it is generally assumed that only part of the genes of the genome are triggered by the conditions applied [25]. In our set up, the part of the network not elicited by the simulated experimental conditions is modeled by adding background genes. These background genes increase the dimension of the data set without being a part of the network to be inferred. Their expression values are assumed to be constitutive but change in a correlated way as a result of the biological noise modeled in the transition functions. In this way, the background network mimics pathways that are not influenced by the simulated conditions. As an alternative to adding a separately chosen background network, one could use the complete source topology, select a limited number of input genes that mimic the external conditions and consider the genes not linked to these input genes as background genes. However, the advantage of selecting subnetworks and adding background genes, is that it allows selecting different topologies instead of using a single network, a property that is required for thorough benchmarking studies.

### Transition functions

After generating the topology, transition functions representing the regulatory interactions between the genes are assigned to the edges in the network. A transition function defines how the mRNA concentration of a gene depends on the mRNA concentrations of each of its input transcription factors [24].

#### Michaelis-Menten and Hill kinetics

Non-linear functions based on Michaelis-Menten and Hill enzyme kinetic equations are used to model gene regulation in steady-state conditions [11,23,26]. As a result of this choice, the generation of expression data scales linearly with the number of genes and therefore allows fast simulation of large networks comprising thousands of genes. Biological noise, corresponding to stochastic variations in gene expression that are unrelated to the applied experimental procedures are modeled by a function based on a lognormal distribution superposed on the kinetic equations.

#### Choosing interactions types

Regulatory interactions between genes can be either activating or inhibiting. When a given gene interacts with more than one regulator, the different regulators can either act independently or exhibit more complex effects on their target genes, such as cooperativity, synergism or antagonism. Different possible interactions are implemented (for a detailed description, see additional file 1: *addendum.pdf*). For each combination of a gene and its regulators, a proper enzyme kinetic equation is selected, depending on the number of activators and repressors and on user-defined settings that control the fraction of complex interactions (see also Section *Generator parameters*).

#### Setting transition function parameters

Choosing realistic parameter settings of these equations is a nontrivial task. Except for a few well characterized networks, no data about the parameters for the Michaelis-Menten and Hill functions is available. Therefore, the value of each parameter is chosen from a distribution that allows a large variation of interaction kinetics likely to occur in true networks (including linear activation functions, sigmoid functions, ...), while avoiding very steep transition functions. An example of the possible interactions is given in Figure 2.

### Sampling data

In this section we describe how a gene expression data set is obtained by simulating the synthetic network under different simulated experimental conditions.

#### Generating gene expression data

When generating data, we assume that the expression of the genes depends on how changes in external conditions trigger the network. External conditions are modeled by choosing a gene set without regulatory inputs and setting their expression level to a different value for each experiment, in a simulated response to changing experimental conditions. Remaining genes without regulatory inputs are assigned a random constitutive expression level.

In a real-world experiment it is possible that input genes show correlated behavior when changing experimental conditions. To account for this, our tool allows positive or negative linear correlation between input genes. Both the number of correlated inputs and the strength of the correlations are user-definable.

The expression levels of the genes in the network are subsequently calculated, as specified by their transition functions, starting from the input genes. For genes involved in cycles, it is possible that not all inputs of their transition function are known during propagation of the values through the network. To model these loops, an approximation compatible with the steady-state transition functions was chosen. Each edge in a cycle is modeled as a regular steady-state interaction and in each simulated experiment, genes that have an undefined input are initially assigned an arbitrary expression value. Calculations for the entire network are then repeated until transient effects have disappeared before generating the output expression values. In case of oscillatory behavior, the expression data is taken from an arbitrary point in the period, mimicking the situation in a real microarray experiment.

#### Adding noise

After sampling from the network, a data set with mRNA expression levels for all genes is obtained for different simulated conditions. All gene expression values are normalized between 0 and 1, where 0 indicates that no transcription occurred and 1 refers to a maximal level of transcription. Besides the biological noise, microarrays are subject to random experimental noise. This experimental noise is added to the simulated microarray data and is approximated by a lognormal distribution [27].

### Generator parameters

To benchmark an algorithm, having access to data sets of an increasing level of complexity is useful. Experience shows that in real data, the difficulty of the structure learning task of an inference problem is influenced to a large extent by the topology of the network to be inferred and by the type of the regulatory interactions present. For example, more data is required to resolve interactions that are not fully exercised [8].

Initial performance testing of an algorithm can be done on rather easy problems (e.g. small, noiseless networks without synergism or cooperativity between regulators). Increasingly difficult data sets can then be generated to further optimize the inference method.

The following parameters controlling the gene network generation and sampling process are user-definable:

• The choice of source network.

• The size of the network in number of nodes.

• The number of background nodes.

• The number of available experiments and samples for each condition.

• The level of stochastic and experimental noise.

• The fraction of complex interactions.

## List of abbreviations

**TRN **Transcriptional regulatory network

**ODE **Ordinary differential equation

**ER **Erdös-Rényi

**SW **Watts-Strogatz or small-world

**AB **Albert-Barabási

**DSF **directed scale free

## Authors' contributions

The first two authors contributed equally to this project. TVdB and KVL wrote the implementation, carried out the simulations and analyzed the data. BN and KM conceived of the study and participated in its design and coordination. PvR participated in the design of the study and helped to draft the manuscript. HM provided the *E. coli *network dataset. All authors contributed to, read and approved the final manuscript.

## Supplementary Material

Additional File 1 Topological measures, types of random networks and interaction types. Click here for file

Additional File 2 Variation of topological measures in function of the number of nodes. Click here for file

Additional File 3 Software implementation of network generator (Java). Click here for file
