# Supplementary material for: SynTReN: a generator of synthetic gene expression data for design and analysis of structure learning algorithms
Source: BMC Bioinformatics. 2006 Jan 26;7:43. doi: 10.1186/1471-2105-7-43 (PMC1373604; doi:10.1186/1471-2105-7-43)
Supplement: Additional File 1 — Topological measures, types of random networks and interaction types. [file 1471-2105-7-43-S1.pdf]

ADDENDUM
SynTReN: a generator of biologically plausible synthetic gene expression data for design and analysis of structure learning algorithms

Tim Van den Bulcke[†1], Koenraad Van Leemput[†2], Bart Naudts[2], Piet van Remortel[2], Hongwu Ma[3], Alain Verschoren[2], Bart De Moor[1]and Kathleen Marchal[*1,4]

[1]ESAT-SCD, K.U.Leuven, Kasteelpark Arenberg 10, B-3001 Heverlee, Belgium
[2]ISLab, Dept. Math. and Comp. Sc., University of Antwerp, Middelheimlaan 1, B-2020 Antwerpen, Belgium
[3]Dept. of Genome Analysis, German Research Center for Biotechnology, Mascheroder Weg 1, D-38124 Braunschweig, Germany
[4]CMPG, Dept. Microbial and Molecular Systems, K.U.Leuven, Kasteelpark Arenberg 20, B-3001 Heverlee, Belgium

Email: Tim Van den Bulcke - tim.vandenbulcke@esat.kuleuven.be; Koenraad Van Leemput - koen.vanleemput@ua.ac.be; Bart Naudts - bart.naudts@ua.ac.be; Piet van Remortel - piet.vanremortel@ua.ac.be; Hongwu Ma - hma2@inf.ed.ac.uk; Alain Verschoren - alain.verschoren@ua.ac.be; Bart De Moor - bart.demoor@esat.kuleuven.be; Kathleen Marchal*- kathleen.marchal@biw.kuleuven.be;

*Corresponding author

†Contributed equally

Topological measures

Not many deterministic and informative topological measures are available [1]. The established measures can be roughly divided into two categories: high-level (global) measures and low-level (local) measures. In order to calculate the high-level property measures (e.g. average path length) one needs to know the whole network, while the low-level properties can be calculated locally (e.g. marginal degree of individual node).

We use both low and high-level topological measures that address different aspects of the network structure. The high-level measures contain network indices such as average clustering coefficient and average path length. The low-level measures are composed of both marginal and bivariate joint degree distributions [1,2].

The *average path length* $\bar{l}$ is defined as follows: Given two genes, let l_{ij} be the length of the shortest path connecting these two genes, following the links present in the network. Depending on the type of network, these can either be directed and/or undirected links. N is defined as the number of nodes in the network. The average path length $\bar{l}$ is defined as:

$$\bar{l} = \frac{2}{N(N-1)} \cdot \sum_{i<j}^{N} l_{ij}$$

The *adjacency matrix* ξ_{ij} indicates an interaction between genes γ_i and γ_j ($\xi_{ij} = 1$) or no interaction ($\xi_{ij} = 0$).

The set of nearest neighbors of a gene γ_i is indicated by $\Gamma_i = \{\gamma_j | \xi ij = 1\}$. *The clustering coefficient* C_i

for this gene is defined as the ratio between the actual number of connections between the genes in Γ_i, and the total possible number of connections, $\frac{|\Gamma_i|(|\Gamma_i|-1)}{2}$.

Formally, the clustering coefficient of the i-th gene C_i is defined as:

$$C_i = \frac{2 \cdot L_i}{|\Gamma_i|(|\Gamma_i| - 1)}$$

where

$$L_i = \sum_{j=1}^{N} \xi_{ij} \cdot [\sum_{k \in \Gamma_i} \xi_{jk}]$$

The (average) clustering coefficient is defined as the average C_i over all genes γ_i:

$$C = \frac{1}{N} \sum_{i=1}^{N} C_i$$

Types of random networks

In [3,4], different types of random graphs are used as a network topology: Erdös-Rényi [5], Albert-Barabási [1] and Watts-Strogatz [6] random graph models. These models and the DSF model of Bollobás [7] will be briefly described. We refer to the literature [1, 5] for further details.

Erdös-Rényi (random network)

As described in [1], a random Erdös-Rényi graph can be defined by a binomial model. We start with N nodes and connect every pair of nodes with probability p. Figure 7 shows a series of 6-node graphs with different p values.

Watts-Strogatz (small-world network)

The model of Watts and Strogatz interpolates between an ordered lattice and a random graph according to the following algorithm (after [1]):

1. Start with a ring lattice with N nodes, where every node is connected to its K nearest neighbors.

2. Randomly rewire each edge with probability p, but avoid self-edges and duplicate edges.

This process introduces long-range edges among the initial short-range edges (see Figure 8) which connect nodes that otherwise would be part of different neighborhoods. These networks have a small-world property: the shortest path between any two nodes is small on average.

Albert-Barabási (scale-free network)

The model of Albert and Barabási is based on two basic principles: *growth* and *preferential attachment*. The graph starts with a small number (m_0) of nodes, and for a series of timestaps, a new node is added each time with a number of edges according to the following rules:

1. Growth: at every time step, we add a new node with m ($< m_0$) edges that link the new node to m different nodes already present in the system.

2. Preferential attachment: the probability p that a new node will be connected to an existing node is proportional to the degree of that node.

Based on these rules, it can be proven that the probability that a node has k edges follows a power law [1,8].

Bollobás (directed scale-free network)
Bollobás et al. [8] present an extension of the Albert-Barabási model [1] for directed graphs. Following the same basic principles, these graphs grow with preferential attachment depending on in- and out-degrees. The resulting in- and out-degree distributions are again power laws, possibly with different exponents.

The model has five parameters ($\alpha, \beta, \gamma, \delta_{in}$ and δ_{out}) and starts with any fixed initial directed graph G_o. At each time step:

- With probability α, add a new vertex v together with an edge from v to an existing vertex w, where w is chosen according to the probability distribution $d_{in}(w) + \delta_{in}$ where $d_{in}(w)$ is the in-degree of w.
- With probability β, add an edge from an existing vertex v to an existing vertex w, where v and w are chosen independently, v according to $d_{out}(v) + \delta_{out}$, and w according to $d_{in}(w) + \delta_{in}$.
- With probability γ, add a new vertex w and add an edge from an existing vertex v to w, where v is chosen according to $d_{out}(v) + \delta_{out}$.

The following equations hold: $\alpha + \gamma > 0$ and $\alpha + \beta + \gamma = 1$.

Interaction types

Equations based on Michaelis-Menten and Hill kinetics have been used to model different types of local interactions between a gene and its parents [4, 9, 10]. In our implementation, all gene expression values are normalized between 0 and 1, where 0 indicates that no transcription occurs and 1 refers to a maximal level of transcription.

The mRNA transcription rate has the form $\frac{\delta r}{\delta t} = v - k_d[r]$ where v is the mRNA production rate, $[r]$ is the concentration of the produced mRNA and k_d is the degradation constant of the mRNA. In steady-state conditions, $\frac{\delta r}{\delta t} = 0$ and $[r] = \frac{v}{k_d}$. The normalization of the expression value is done by applying the following boundary conditions:

- $r = 1$ when $[A_i] = 1$ and $[I_j] = 0, \forall i, j$
- $r = 0$ when $[A_i] = 0$ and $[I_j] = 1, \forall i, j$

where $[A_i]$ are the concentrations of the activators, $[I_j]$ are the concentrations of the inhibitors.

Intuitively, it is easy to see that, given a fixed production rate, a lower degradation constant will lead to a higher absolute number of mRNA copies in the cell, because it takes longer to reach equilibrium. However, because of the rescaling step described above, the effect of the degradation constant is canceled. The data produced by our tool can therefore be compared to adequately pre-processed and scaled expression data. Scaling of expression data will bring the expression values of different genes into the same range and is typically a first step in analysis of expression profiles because it allows detection of qualitatively similar expression profiles between genes that have a different absolute range of expression values.

A distinction has been made between different types of interactions (including cooperative, competitive, non-competitive and synergistic interactions). In the general case for N regulators, an empirical generalization is made to model Hill kinetic interactions, similar to [4]. No interactions such as competitivity, non-competitivity and synergism are modeled for the general case. A derivation of such an overall rate law is outside the scope of this work. The general steady-state equation for N regulators (P activators, Q inhibitors) is given by:

$$v = \frac{V_{0,max} + \sum_{i=1}^{P}(\frac{A_i}{K_i})^{n_i^{act}} \cdot \prod_{j=1}^{P \neq i}(1+(\frac{A_j}{K_j})^{n_i^{act}}) \cdot V_{i,max}}{\prod_{i=1}^{P}(1+(\frac{A_i}{K_i})^{n_i^{act}}) \cdot \prod_{j=1}^{Q}(1+(\frac{I_j}{K_j})^{n_j^{inh}})}$$

The exponents n_i^{act} and n_j^{inh} are the Hill constants for the specific activators and inhibitors. Hill constants are integer and $n_i^{act} \geq 1$, $n_j^{inh} \geq 1, \forall i, j$.

Specialized interaction types were derived for cases with two regulators and are given by the equations below.

One activator and one repressor, competitive:

$$v = \frac{V_{0,max} + V_{1,max} \cdot \frac{A}{K_a}}{1 + \frac{I}{K_i} + \frac{A}{K_a}}$$

One activator and one repressor, non-competitive:

$$v = \frac{V_{0,max} + V_{1,max} \cdot \frac{A}{K_a}}{(1 + \frac{I}{K_i}) \cdot (1 + \frac{A}{K_a})}$$

Two activators, synergism:

$$V_{3,max} = \beta \cdot (V_{1,max} + V_{2,max})$$

$$v = \frac{V_{0,max} + \frac{V_{1,max} \cdot A_1}{K_{1a}} + \frac{V_{2,max} \cdot A_2}{K_{2a}} + \frac{V_{3,max} \cdot A_1 \cdot A_2}{K_{1a} \cdot K_{2a}}}{1 + \frac{A_1}{K_{1a}} + \frac{A_2}{K_{2a}} + \frac{A_1 \cdot A_2}{K_{1a} \cdot K_{2a}}}$$

where β denotes the degree of synergism between the two activators.

The parameters of all these equations are chosen from predefined distributions, and are chosen in a range that allows for a variation in possible interaction kinetics that are likely to occur in true networks (including linear activation functions, sigmoid functions, ...), while avoiding very steep transition functions. For example, the distribution for the values of K_h is centered around 0.5. For K_h equal to 0.5, the transcription rate of the target of a regulator is at 50% of its maximal transcription rate if the regulator is at 50% of its maximal transcription rate. For high and low values of K_h, the relation between regulator and its target is steeper.

References

1. Albert R, Barabási A: **Statistical mechanics of complex networks**. *Reviews of Modern Physics* 2002, **74**:47–97.
2. Zhu D, Qin ZS: **Structural comparison of metabolic networks in selected single cell organisms**. *BMC. Bioinformatics* 2005, **6**:8.
3. Knüpfer C, Dittrich P, Beckstein C: *Artificial Gene Regulation: A Data Source for Validation of Reverse Bioengineering*, Akademische Verlagsgesellschaft Aka, Berlin. 2004 :66–75.
4. Mendes P, Sha W, Ye K: **Artificial gene networks for objective comparison of analysis algorithms**. *Bioinformatics* 2003, **19**:II122–II129.
5. Erdös P, Rényi A: **On random graphs**. *Publ. Math. Debrecen* 1959, **6**:290–297.
6. Watts DJ, Strogatz SH: **Collective dynamics of 'small-world' networks**. *Nature* 1998, **393**:440–442.
7. Bollobas B, Borgs C, Chayes C, Riordan O: **Directed scale-free graphs**. *Proceedings of the 14th ACM-SIAM Symposium on Discrete Algorithms* 2003, :132–139.
8. Bollobás B: *Random graphs.* Academic Press, New York 1985.
9. Fersht A: *Enzyme structure and mechanism*, Volume 2. W.H. Freeman and Company, New York 1985.
10. Hofmeyr JH, Cornish-Bowden A: **The reversible Hill equation: how to incorporate cooperative enzymes into metabolic models**. *Comput. Appl. Biosci.* 1997, **13**:377–385.
