# Supplementary figures and images for: SynTReN: a generator of synthetic gene expression data for design and analysis of structure learning algorithms
Source: BMC Bioinformatics. 2006 Jan 26;7:43. doi: 10.1186/1471-2105-7-43 (PMC1373604; doi:10.1186/1471-2105-7-43)

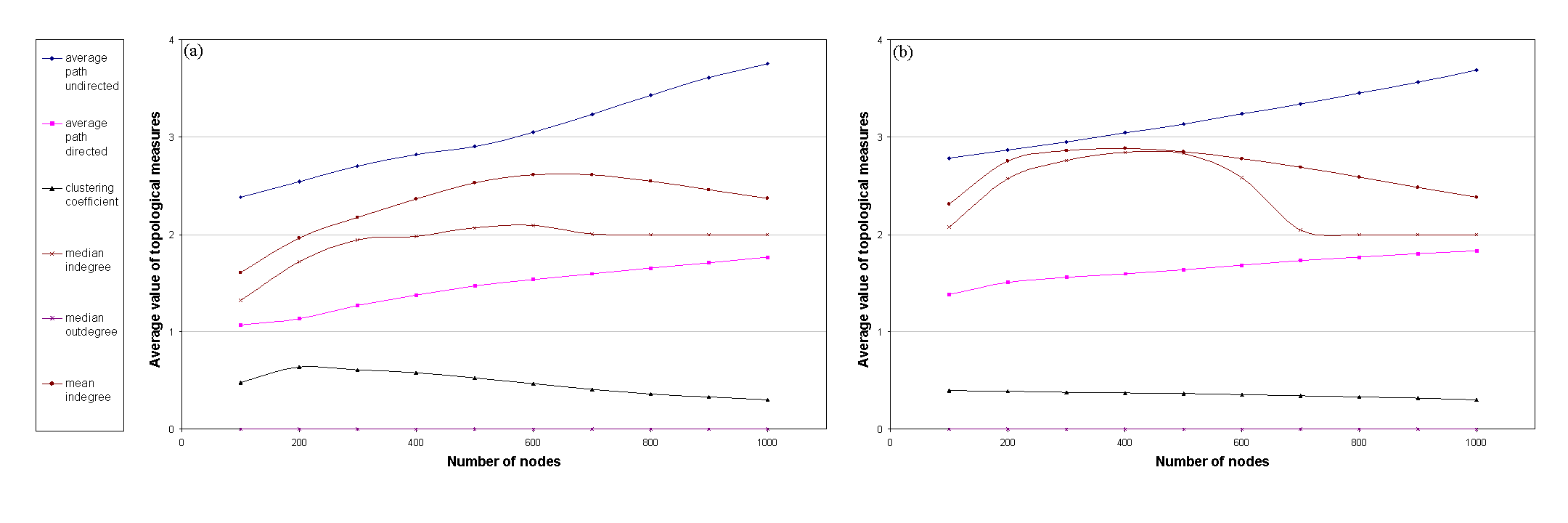

Supplement: Additional File 2 — Variation of topological measures in function of the number of nodes. [file 1471-2105-7-43-S2.png]
